# Nanomaterials promote the fast development of electrochemical MiRNA biosensors

**DOI:** 10.1039/d3ra08258j

**Published:** 2024-06-04

**Authors:** Ruizhuo Ouyang, Ying Huang, Yuanhui Ma, Meina Feng, Xi Liu, Chongrui Geng, Yuefeng Zhao, Shuang Zhou, Baolin Liu, Yuqing Miao

**Affiliations:** a Institute of Bismuth and Rhenium Science, University of Shanghai for Science and Technology Shanghai 200093 China ouyanrz@usst.edu.cn; b Cancer Institute, Tongji University School of Medicine Shanghai 200093 China; c School of Health Science and Engineering, University of Shanghai for Science and Technology Shanghai 200093 China

## Abstract

Cancer has become the leading cause of death worldwide. In recent years, molecular diagnosis has demonstrated great potential in the prediction and diagnosis of cancer. MicroRNAs (miRNAs) are short oligonucleotides that regulate gene expression and cell function and are considered ideal biomarkers for cancer detection, diagnosis, and patient prognosis. Therefore, the specific and sensitive detection of ultra-low quantities of miRNA is of great significance. MiRNA biosensors based on electrochemical technology have advantages of high sensitivity, low cost and fast response. Nanomaterials show great potential in miRNA electrochemical detection and promote the rapid development of electrochemical miRNA biosensors. Some methods and signal amplification strategies for miRNA detection in recent years are reviewed herein, followed by a discussion of the latest progress in electrochemical miRNA detection based on different types of nanomaterial. Future perspectives and challenges are also proposed for further exploration of nanomaterials to bring breakthroughs in electrochemical miRNA detection.

## Abstract 2

Representation of traditional detection methods and amplification strategies for the detection of miRNA, and the application of different types of nanomaterials in electrochemical miRNA biosensors.

## Graphical abstract

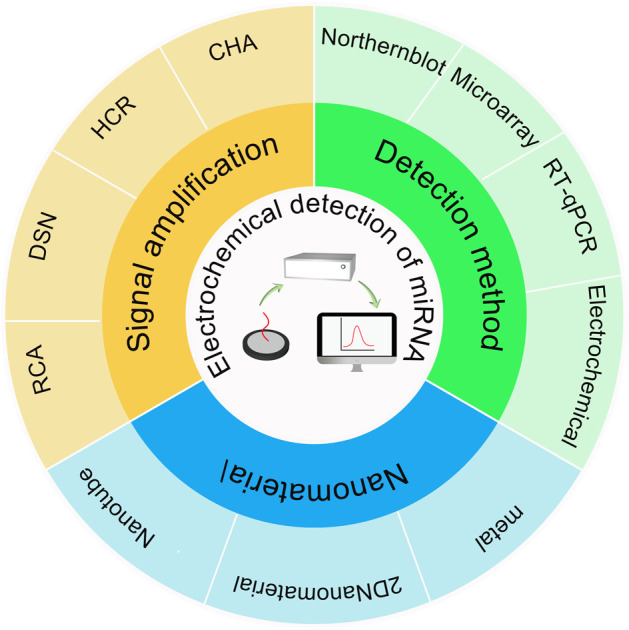

## 1 Introduction

It is well known that cancer not only consumes a lot of medical resources but also poses a great threat to human life. With continuous progress made in the treatment of cancer, early diagnosis of cancer has been proven to be an integral part of the treatment of cancer. Compared with late-diagnosed cancer patients, people with early diagnosis of cancer have an improved chance of survival and provide doctors with more treatment time.^1–3^ However, the complexity and diversity of cancer are still a great obstacle to the development of new diagnostic methods. At present, cancer screening is carried out mainly using non-molecular techniques such as low-dose computed tomography, gastroscopy, and protein biomarkers, which have low sensitivity and specificity.^4–6^ As a fluid biopsy analyte, miRNAs can be present in body fluids such as saliva, plasma and urine^7^ and are associated with various diseases including cancer. Because of their non-invasive characteristics, they have been regarded as ideal biomarkers for early clinical diagnosis of cancer.

MiRNA was first discovered in *Caenorhabditis elegans* by Lee *et al.* in 1993. It is about 22 nucleotides long and belongs to a class of endogenous non-protein-coded single-stranded small RNA molecules. It can regulate gene expression at the translational or post-transcriptional level and plays an important role in almost all known cellular processes, including cell cycle regulation, differentiation, apoptosis, organ development and immune processes. MiRNA remains highly stable under extreme conditions that can lead to the degradation of most RNAs.^8^ In the past few years, miRNAs have been well studied in different types of cancer and other diseases. The abnormal expression of miRNAs, whether up-regulated or down-regulated, clearly contributes to the important biological process abnormalities, which are closely related to a variety of diseases including cancers,^9–14^ lung diseases,^15,16^ cardiovascular diseases,^17,18^ and autoimmune diseases.^19,20^ Considering the availability and stability of miRNAs in body fluids, level monitoring of miRNAs is an effective method for early diagnosis of the disease through invasive sample collection. As a result, it has become an ideal biomarker for disease diagnosis and detection, thus greatly triggering the fast development of the detection of miRNAs. The traditional detection methods of miRNAs include Northern blotting,^21^ real-time quantitative polymerase chain reaction (qRTPCR),^22^ deep sequencing,^23^ and *in situ* hybridization.^24^ Most of these detection techniques are based on nucleic acid detection technology, which relies on optical detection. However, these methods are usually time-consuming and high-cost. Therefore, it is greatly significant to develop an efficient, simple and sensitive detection method. Among the various techniques used to detect miRNA, biosensors, especially electrochemical detection technology, have proved to be a potent and promising strategy due to their low cost, easy miniaturization, fast response, and high sensitivity.

Currently, more and more nanomaterials have been used to develop electrochemical sensors, including Au,^25^ Pt,^26^ carbon nanomaterials,^27^ MoS_2_,^28^ CeO_2_,^29^*etc.*, because of their unique properties, especially high specific surface area, which is good for the effective immobilization of biologically active molecules. The high electrical conductivity, magnetism and catalytic activity of nanomaterials are very important and are essential for biosensors with high sensitivity to detect a variety of analytes, such as cancer, viruses and miRNA biomarkers.^30–32^ This review introduces several detection methods and strategies for miRNAs and the recent application of nanomaterials in the electrochemical biosensors for miRNA detection.

## 2 Methods for miRNA detection

As a non-invasive blood biomarker, miRNA exists in tissues and body fluids and is relatively stable and easy to detect. Given the important role of miRNA in the early diagnosis of some diseases, including cancer, it is more important to develop detection methods that can achieve specificity and sensitivity. Because of the availability and stability of miRNA in body fluids, it is recommended to detect miRNA by analyzing biological body fluids. The concentration of miRNA is between femtomolar (fM) and picomolar (pM).^33^ In the past few decades, many new miRNA detection methods have been developed. The methods for the detection of miRNA can be divided into two categories: traditional detection methods and new alternative detection methods. The most commonly used classical techniques are nucleic acid detection methods, including Northern blotting, microarray, and quantitative real-time PCR (RT-PCR). The new detection technologies can be divided into two categories: optical biosensor platforms and electrochemical biological platforms,^34^ such as surface plasmon resonance, photochemistry, electrochemistry, and electrochemiluminescence. Herein, we discuss and summarize some conventional detection methods and alternative detection methods, and analyze their advantages and disadvantages.

### 2.1 Northern blot

**Fig. 1:**
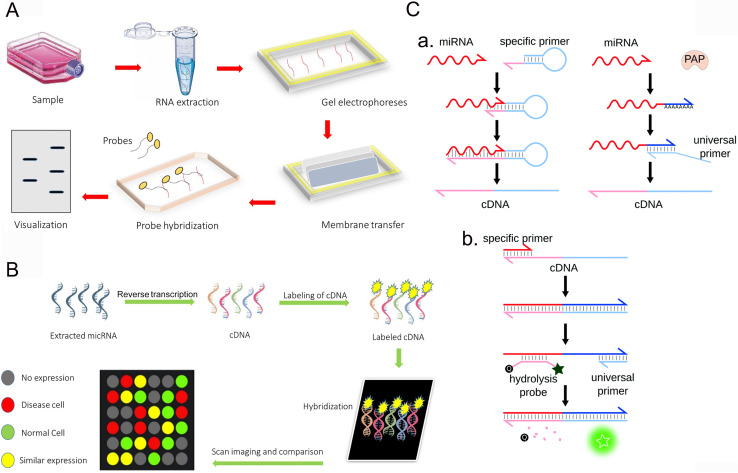
Conventional methods for the detection of miRNA. (A) Northern blot.^44^ Copyright 2022, Elsevier. (B) Microarrays.^44^ Copyright 2022, Elsevier. (C) RT-qPCR.^45^ Copyright 2021, Royal Society of Chemistry.

Northern blot is a common method for RNA detection based on hybridization (Fig. 1A). It is one of the earliest methods for miRNA analysis.^21^ To date, it is still the gold standard of miRNA expression profile analysis and is widely used. This detection method is an experimental method for nucleic acid hybridization by transferring the sample RNA digested by restriction endonuclease from electrophoretic gel to nitrocellulose filter membrane or other chemically modified active filter paper. The basic principle is that RNA is separated according to size and detected on the membrane by hybridization probes of base sequences complementary to all or part of the target miRNA. However, this technique takes a long time and is not sensitive enough.^35,36^ A large number of manual operations are designed for analysis, and only one miRNA probe is hybridized with one RNA blotting at a time, so this detection method is not suitable for detecting a large number of different miRNAs; rich biomaterials are needed. However, mature miRNA molecules are very short and account for a relatively low proportion of total RNA, so this technique can only provide semi-quantitative results. Northern imprinting is not only time-consuming but also time-consuming, and it takes more than one working day for miRNA detection, which leads to the risk of signal diffusion. Some laboratories have developed new Northern imprinted versions with higher sensitivity and less total detection time. Given the low sensitivity and specificity of Northern blotting, an alternative DNA probe was developed. This disadvantage is greatly improved by the use of locked nucleic acid (LNA) probes. In addition, the method of marking sequence is also used to improve the sensitivity, and the short RNA sequence is marked. For example, its short RNA sequence is labeled with 3-digoxin, which is revealed using anti-digoxin antibodies and can also be used to identify targets.^37^ Although these strategies improve the sensitivity, the improved Northern imprinting technique is expensive and time-consuming, so it is unlikely to be used as a conventional diagnostic method.

### 2.2 Microarray

Microarray technology is also a method of hybridization sequencing (Fig. 1B). The principle of microarray is to use high-density fluorescence-labeled probes to hybridize with RNA samples to analyze the target molecules by observing the hybridization signals. The expression map was obtained by fluorescence scanning, and the expression of miRNA was analyzed with the help of the corresponding software.^38^ By measuring the expression level of miRNA in a specific process, one can analyze and understand the expression regulation mechanism of miRNA and the expression of genes regulated by miRNA. Because all available miRNA sequences can be included in the design of probes, microarrays can screen a large number of miRNAs at the same time. This strategy can be used for miRNA maps. However, microarray technology has low sensitivity and weak specificity in the detection of miRNAs, so it is designed for recognition and cannot achieve the quantitative detection of miRNAs. It is mainly limited by several inherent characteristics of miRNA, including the short length of miRNAs, low abundance of some miRNA, and some different miRNAs belonging to the family sharing similar sequences with little difference.^39^ It is not easy to amplify these short RNA targets. To overcome these problems, several nucleic acid analogues have been sent out, showing more favorable hybridization characteristics. Another disadvantage of microarray technology is that the manufacturing cost of microarray chips is also very high and cannot be routinely implemented in basic and clinical research laboratories.

### 2.3 Reverse transcriptase-polymerase chain reaction (RT-qPCR)

RT-qPCR is an amplification method with high sensitivity, large dynamic range and simple operation (Fig. 1C). First, the target miRNA was transformed into cDNA by reverse transcription, and then the expression profile was obtained by polymerase chain reaction and fluorescence scanning. Reverse transcription usually uses stem-loop primers, poly(T) tailing or gene-specific primers (GSP) containing tail sequences. Chen *et al.* introduced a stem-loop primer for miRNA transcription.^40^ Because its stem accumulation ability and space share exceed those of the standard linear primers, it promotes the thermal stability of the heteroduplex and improves the sensitivity and stability. Another method is to add poly(T) or poly(A) connectors to miRNA primers.^41–43^ At present, there are two main methods for detecting miRNA, namely, the RT-qPCR, Taq-Man probe method, and the SYBR green fluorescence dye method. Although quantitative real-time PCR requires fewer samples than Northern imprinting analysis and microarray and significantly improves the dynamic range and sensitivity, this technique still faces challenges in the detection of miRNA. Due to the problems of false positives and difficulty in primer design, its application is limited. Relying on the connection of multiple steps, accurate and repeatable results can be obtained, and many factors need to be considered, which put forward higher requirements for the laboratory.

## 3 Electrochemical biosensors

Although the traditional detection methods can also be applied to detection, they all have their own limitations. Compared with traditional detection methods, the electrochemical biosensor is simpler and more effective for the detection of miRNA. Biosensors can be used to detect whether the miRNA expression of a gene is abnormal to get an indication of whether the disease can occur.^46^ Compared with the optical biosensor, the electrochemical sensor uses the electrochemical instrument as the signal converter to connect the concentration change of the tested component with the electrical signal to provide real-time information about the chemical component to be measured in the tested system, and the target miRNA can be qualitatively and quantitatively analyzed through the change in the electrical signal.^47,48^ The electrochemical detection method has the advantages of low cost, simple structure, high sensitivity, good stability, and easy miniaturization. It can be used for the real-time detection of nucleotide hybridization reactions and time-resolved measurement. The main problem in the design of electrochemical sensors is to find a method to generate the electrical signal for detection. Typical biosensors are composed of fixed electrodes and electroactive hybridization indicators, and short single-stranded nucleotide probes are fixed on the fixed electrodes. The complementarity of hybridization between miRNA and single-stranded probes in cells or tissues affects the activity of the biosensors, which depends on the selection of probes and hybridization types. The detection of miRNA by an electrochemical sensor can be divided into two categories: labeled detection and unlabeled detection. Signal amplification is a key problem in the construction of miRNA electrochemical biosensors. Combined with a variety of new nanomaterials with excellent properties, the application of an effective signal amplification strategy is of great significance for realizing the specific and sensitive detection of miRNAs.

### 3.1 Nanomaterials for enhancing the sensitivity of electrochemical biosensors

Nanomaterials, including metallic nanomaterials,^49^ 2D nanomaterials,^50^ and nanotubes,^51^ have significantly improved the sensitivity of electrochemical biosensors. Metallic nanomaterials are more widely used for their excellent electrical conductivity as well as catalytic properties. Particularly, gold nanoparticles (Au NPs) can bind to capture probes through covalent bonding,^52^ which is the basis of many nanomaterials for the preparation of electrochemical biosensors. 2D nanomaterials such as graphene^53^ and MXene^54^ have high specific surface area, excellent electrical conductivity, and easy modification, which can provide more active sites and thus promote the improvement of sensitivity. Target-induced redox signal amplification has been used for electrochemical biosensors in the determination of circulating miRNA-21 in the cerebrospinal fluid of patients with medulloblastoma,^55^ where only Au NPs were used to fix the capture probe. Upon combination with a long sequence rich in guanine, many methylene blue indicators could specifically adsorb to produce amplified electrochemical redox signals. Without the use of nanocomposites, the sensitive detection of the target is realized simply and efficiently. Also, a graphite diyne self-powered biosensor platform was developed for the dual-mode detection of miRNA by integrating T7 exonuclease and DNA walker-induced rolling ring amplification.^56^ With the excellent conductivity and large specific surface area of graphite diyne as the electrode substrate, the Au NPs-anchored capture probe generated an enhanced sensing signal when combined with T7 exonuclease-assisted DNA walker recognition and amplification, greatly improving the sensitivity. Therefore, designing novel electrochemical biosensors based on the properties of nanomaterials will further reduce the detection limit, broaden the detection range, and achieve sensitive detection.

### 3.2 Nanomaterials for improving the selectivity of electrochemical biosensors

To improve the selectivity of electrochemical biosensors, it is necessary to achieve specific recognition of the detection target. Nanomaterials that can be easily modified and functionalized are particularly important and preferred, among which, 2D nanomaterials have the unique advantage of being easily modified with other nanoparticles, such as precious metal nanomaterials^57,58^ to form nanocomposites to achieve specific recognition and improve selectivity. For example, novel electrochemical biosensors for breast cancer based on carbon nanofibers,^59^ metal–organic skeletons, and magnetic graphene oxide nanocomposites can anchor larger targets using the large specific surface area of nanocomposites, since it could be modified with iron atoms to prevent single-chain shedding, achieve specific adsorption and improve selectivity. Au NPs significantly improve selectivity due to the effect of specific covalent bond binding. After being modified to improve electrical conductivity or catalytic activity, nanomaterials can achieve the specific detection and recognition of different targets due to the improved selectivity of electrochemical biosensors. Another electrochemical biosensor for the simultaneous detection of miRNA in breast cancer screening was developed^60^ using porous hollow silver Au NPs with large surface area as conjugated medium nanoparticles to capture DNA, and metal ions as signal markers to detect a variety of miRNA and amplify signals. Combining nanomaterials with metal particles that could generate detectable signals not only improves the selectivity, it also facilitates the detection of multiple miRNAs.

## 4 Signal amplification and amplification strategy

The key point of the nanomaterials-mediated electrochemical detection of miRNAs is signal amplification. Since the level of miRNAs is low in tissues or cells, several amplification strategies are needed for miRNA electrochemical biosensors *via* combining nanomaterials to improve detection sensitivity. The electrochemical miRNA biosensors recognize the target miRNAs using a capture probe. However, hybridization alone does not generate a higher signal. Signal amplification strategies can further enhance the detection signal towards the target miRNA, thus improving the sensitivity and detection limit.

### 4.1 Rolling circle amplification (RCA)

**Fig. 2:**
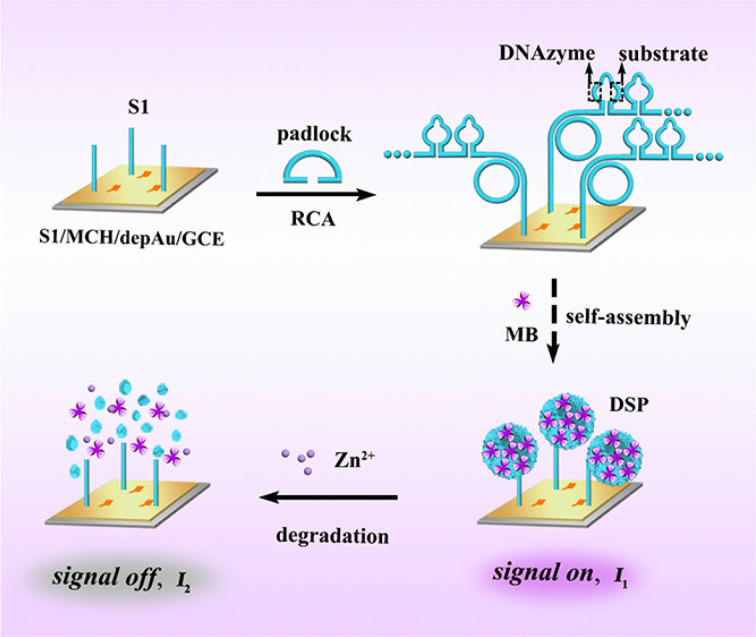
RCA is triggered to generate functional DNA nanospheres encoded by DNAzyme and substrate sequences for loading a large number of signal tags with significant electrochemical signals.^63^ Copyright 2022, American Chemical Society.

RCA technology is a nucleic acid amplification technology established by learning from the rolling loop replication mode of DNA molecules of circular pathogenic microorganisms in nature. It is a DNA amplification technology that occurs at constant temperature. In the RCA reaction, when there is a circular DNA template and polymerase, through the action of DNA polymerase, the circular DNA is used as the template to replicate and finally, a long single-stranded DNA (ssDNA) of the repetitive sequence complementary to the circular DNA template is formed. According to the number of primers, RCA can be divided into linear amplification, hyperbranched amplification and cascade amplification.^61^ The traditional linear RCA amplification has gradually developed into exponential amplification, which can be achieved by using additional primers complementary to the original repetitive DNA. Shang *et al.* extended the RCA process to the hyperdendritic RCA (HRCA) detection of bioactive substance sensing systems.^62^ Because RCA amplification technology has the advantages of simple operation and flexible application, it is often preferred to be used in combination with other amplification methods (Fig. 2). For example, Kong *et al.* proposed a novel proportional electrochemical biosensor with methylene blue (MB) as the only signal label for the detection of miRNA-21 under RCA cascade amplification.^63^ In this sensor, the target miRNA-21 can be converted into a large number of simulated target DNAS1 and cofactor Zn^2+^ by target-induced recycling and acid dissolution, respectively. When S1 is modified to the electrode by Au–S bonding, RCA is triggered by S1 to form functional DNA nanospheres (DSP), which are used to load a large number of signal tags of MB with significant electrochemical signals (signal on). Cofactors mediate the non-violent DNAzyme catalytic cracking of DSP to release MB sharply, resulting in an electrochemical response. With the help of innovative target-induced double-signal amplification and RCA reaction, the effect of double-signal amplification is achieved and the sensitivity of detection is improved.

### 4.2 Duplex-specific nuclease amplification (DSN)

**Fig. 3:**
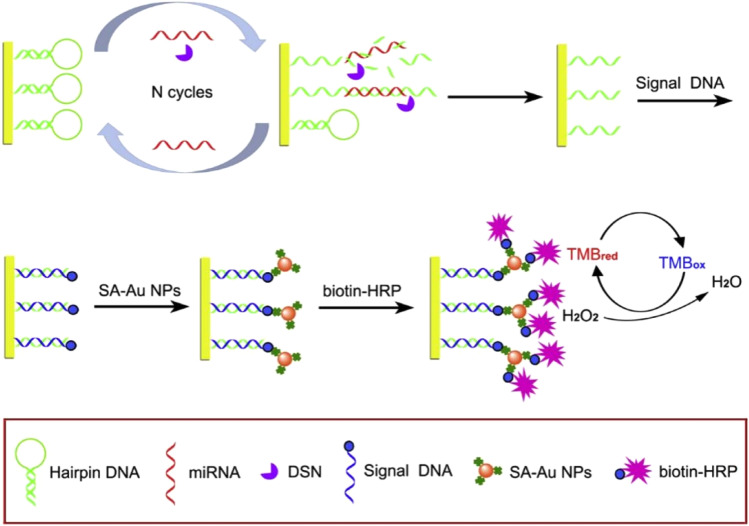
Schematic illustration of the electrochemical miRNA biosensor based on DSN-assisted target recycling followed by the Au NPs and HRP enzymatic signal amplification strategy.^64^ Copyright 2019, Elsevier.

DSN is a thermostable nuclease isolated from the Kamchatka crab. The nuclease can specifically degrade dsDNA or the DNA–RNA duplex and has no activity against single-stranded oligonucleotides or dsRNA. It is possible to construct new miRNA biosensors. This principle is mainly based on the recovery of target miRNA with the assistance of DSN. Through target recovery, a small amount of miRNA can cause a large number of “signal molecules” to be released, thus amplifying the signal. Based on this property, Zhang *et al.* developed a triple signal electrochemical biosensor (Fig. 3) to detect miRNA-21.^64^ Triple-signal amplification is realized by DSN-assisted target recovery alloy NPs and HRP enzymatic signal amplification. The biosensor can distinguish similar miRNA with only one base difference. In the absence of target miRNA, DSN has no activity against hairpin DNA (hDNA). When the target miRNA is incubated with the biosensor, miRNA can hybridize with hDNA to form a DNA/RNA double strand. Then, DSN can recognize and selectively cut most of the double strands, thus causing the release of the target miRNA. The released miRNA can be used for the next loop. The signal is amplified effectively by exponential amplification with the aid of DSN. The alloy nanoparticles have good enzyme activity, high specific surface area/volume ratio, well-controlled surface properties,^65^ the catalytic activity of the HRP enzyme, and the detection limit is as low as 43.3 aM.

### 4.3 Hybrid chain reaction (HCR)

**Fig. 4:**
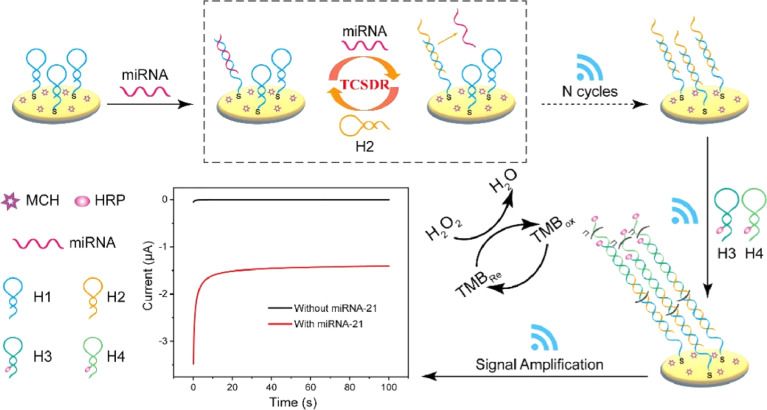
Schematic illustration of the electrochemical biosensor based on triple signal amplification.^67^ Copyright 2023, Elsevier.

The hybrid chain reaction (HCR) refers to the process in which two stable coexisting hairpin DNA are opened and alternately hybridized to form a long double-stranded DNA (dsDNA) by adding the DNA initiation chain under mild conditions.^66^ Compared with the above amplification techniques, the assistance of specific enzymes is not needed. Recently, several strategies have been proposed for miRNA biosensors based on HCR amplification. Fig. 4 shows the electrochemical detection of miRNA based on triple signal amplification based on the hybrid chain reaction.^67^ The capture hairpin probe is connected to the electrode surface by the Au–S bond. HCR amplification is triggered in the presence of target miRNA, and target miRNA-21 is released by strand replacement reaction. The released miRNA-21 constantly reacts with H1 to start the next cycle. At this time, the biosensor achieves the first signal amplification. The formed H2–H1dsDNA has a starting sequence that can trigger the HCR response. The ultra-long dsDNA-labeled HRPH3 and H4 were then successfully assembled on the electrode surface by HCR reaction, and the secondary signal amplification was obtained. HRP loaded on dsDNA can effectively catalyze the mixture of H_2_O_2_ and TMB, causing the third signal amplification, and the detection limit is as low as 0.14 aM.

### 4.4 Catalytic hairpin assembly (CHA)

**Fig. 5:**
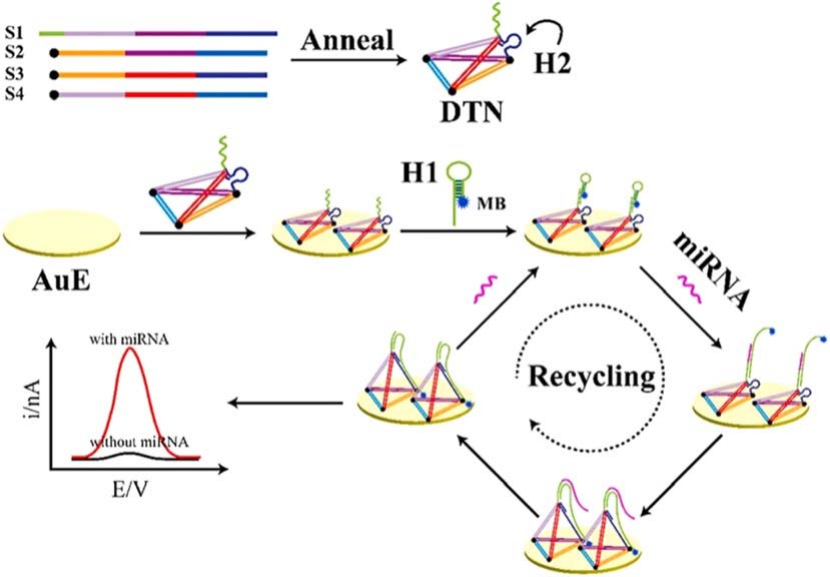
Scheme showing the electrochemical biosensor for miRNA detection based on *in situ* CHA-actuated DTN interfacial probes.^69^ Copyright 2021, Elsevier.

CHA is a non-enzyme signal amplification method. CHA can initiate two designed hairpin DNA to form double strands in the current target sequence through a foothold-mediated chain replacement mechanism, thus achieving high sensitivity and the selective detection of detection targets.^68^ CHA has been widely used in the electrochemical analysis of miRNA because of its simple design, low background and high turnover. Fig. 5 shows a novel biosensor platform based on the *in situ* catalytic hairpin assembly to drive the DNA surface interface probe for the ultra-sensitive detection of tumor biomarker miR-141.^69^ Firstly, four single-stranded DNA were annealed to form a DNA tetrahedral nanostructure (DTN). The DTN probe with three mercaptan-modified vertices was fixed on the electrode surface by Au–S bond interaction. The stem-loop hairpin structure (H2) was embedded at one of the edges of the DTN, and the capture sequence was embedded at the top vertex. Then, the H1 labeled with MB can be linked to DTN by hybridization with the capture sequence. H1 and H2 are carefully designed as fuel chains for CHA reactions and are assembled into nanostructures. In the presence of the target, the interaction between H1 and H2 is triggered, which increases the possibility of direct collision between MB and the electrode surface and promotes electron transfer. At the same time, the target miRNA is released to unfold another loop and amplify the signal. The sensor shows excellent anti-interference ability and a low detection limit of 0.32 fM in the actual sample.

These nucleic acid amplification strategies have helped nanomaterials to facilitate the development of the electrochemical detection of miRNAs, and some strategies combined with nanomaterials can achieve signal amplification. However, the complex amplification steps and the design of amplification strategies are still problems that need to be solved, and concise and effective amplification strategies are constantly being explored.

## 5 Advantages and disadvantages of biosensors based on nanomaterials for the electrochemical detection of miRNAs

With the rapid development of nanotechnology, nanomaterials show great advantages in the fields of medical imaging, disease diagnosis, drug release and gene therapy. In the field of biosensors, nanomaterials are good sensing elements due to their unique optical, electrical, magnetic, mechanical and chemical properties. Nanomaterials-modified biosensors for electrochemical detection of miRNA have the advantages of high sensitivity, high specificity, simple operation, high interference resistance, and easy portability.^70^ The combination of nanomaterials with electrochemical biosensors improves the denaturation of enzymes and other biophilic agents caused by previous immobilization instability.^71^ However, there are some shortcomings in the application of nanomaterials in the electrochemical detection of miRNA sensors. Some nanomaterials are potentially biotoxic and may be agglomerated and oxidized during the process of use, which affects the stability of the sensors. Moreover, the difference in synthesis periods may also affect the performance of the sensors due to the complexity of the synthesis process of nanomaterials. However, with the development of nanotechnology, a variety of new nanomaterials are constantly being explored, and all these problems will be gradually solved. There are few electrochemical biosensors based on pure nanomaterials. Because the sensors for miRNA need to capture miRNA, the capture probe needs to be fixed. Most sensors use Au–S bonds to fix the capture probe on the electrode surface but the application of pure gold nanomaterials is far from meeting the sensitive and specific detection requirements we need. It needs to be combined with other nanomaterials to improve the performance. Compared with simple nanomaterials, nanocomposites are considered ideal in both conductivity and catalytic activity. Table 1 compares the differences between electrochemical miRNA biosensors based on different nanomaterials.

**Table 1:** Comparison of electrochemical miRNA biosensors based on different kinds of nanomaterials

Types of nanomaterials	Advantages and disadvantages	Nanometer material	MiRNA	Technique	Detection limit	Linear range	Ref
Metal nano-materials	Rich surface and interface, high conductivity and good catalytic activity. High cost and potential biotoxicity	SA-PPy/AuNPs	miRNA-21	SWV	0.34 fM	1 fM to 1 nM	52
		AuNPs/CNNS	miRNA-21	SWV	2.9 fM	10 fM to 1 nM	25
		AuNPs and AgNPs	miR-106a	LSV	0.62 fM	1 fM to 1 pM	107
		AgNPs	miRNA-21	SWV	0.4 fM	1 fM to 200 pM	108
2D nano-materials	Large specific surface area, easy modification, and good electrical conductivity. Complicated synthesis and easy aggregation	AuNPs/rGO	miRNA-21	DPV	5 fM	10 fM to 2 pM	109
		MXene–MoS_2_	miRNA-21	SWV	26 fM	100 fM to 100 nM	110
		AuNPs/GP/PPy	miRNA-21	DPV	0.02 fM	0.001 pM to 1.0 nM	111
		AuNPs/PtNPs/GO	miRNA-21	DPV	1 fM	1 fM to 1 μM	112
Nanotube materials	Large specific surface area and good electrical conductivity. Complicated synthesis, difficult size control and high preparation cost	SWCNT/denAu	miRNA-21	DPV	0.01 fM	0.01 fM to 1 μM	113
		Shortened and acidified MWCNTs with AuNPs	miRNA-21	DPV	0.03 pM	0.1–12 000 pM	114
		CNTs, AuNPs	miR-21	EOCV	2.7 aM	0.1–1000 fM	115

### 5.1 Application of electrochemical miRNA biosensors based on metallic nanomaterials

Metal nanomaterials have excellent properties such as high electrical conductivity and good catalytic activity, in addition to abundant surfaces and interfaces, which make them attractive materials for the electrochemical detection of miRNA sensing systems, leading to their wide use in the field of electrochemical sensors. In miRNA electrochemical sensing and detection systems, metal nanomaterials are generally modified on the electrode surface to fix the probe or improve the conductivity, and can also be used as a signaling probe or other signaling-active molecule carrier.

**Fig. 6:**
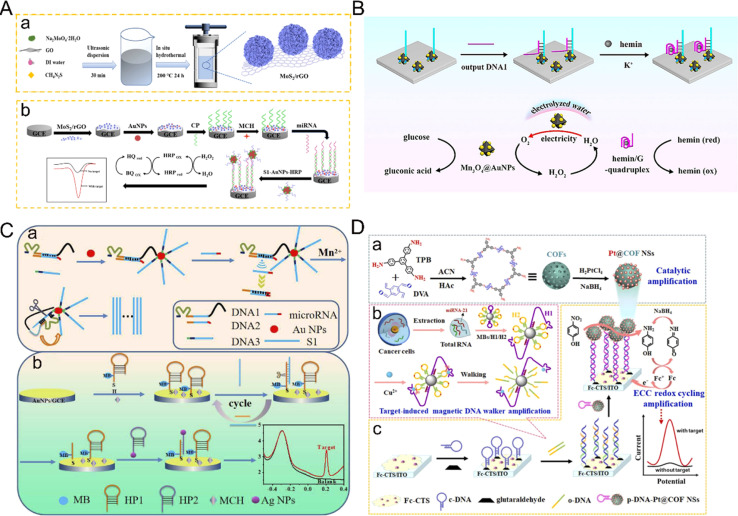
(A) Schematic diagram of miRNA-21 detection based on MoS_2_–rGO and the HRP strategy.^73^ Copyright 2022, Springer Nature. (B) The mimic enzyme cascade catalytic mechanism between the hemin/G-quadruplex DNAzyme with peroxidase-like activity and bifunctional Mn_3_O_4_@AuNPs with glucose oxidase-like activity and self-supplied O_2_ property.^74^ Copyright 2024, Elsevier. (C) Schematic diagram of the ratio of the electrochemical biosensor based on walker amplification and Ag NPs for the detection of miRNAs.^80^ Copyright 2022, Elsevier. (D) Schematic illustration of the Pt@COFNSs biosensor for sensitive miRNA-21 detection.^83^ Copyright 2023, Elsevier.

Au NPs are not only easy to synthesize but also have excellent affinity for various ligands, and low biological toxicity, so they are widely used in biosensors. The strong interaction between mercaptan and gold provides the basis for many nucleic acid biosensors, in which mercaptan-derived nucleic acid fragments are used to modify AuNPs.^72^ The combination of Au NPs and other nanoparticles to improve performance has been a common method in recent years. Dong *et al.* reported a novel sandwich miRNA biosensor based on graphene oxide, which combined three-dimensional flower MoS_2_ and AuNPs with HRP enzyme signal amplification^73^ (Fig. 6A). Firstly, flower-like MoS_2_/rGO nanocomposites with high specific surface area, unique three-dimensional structure and uniform dispersion were synthesized by a one-step hydrothermal method as electrode modification materials. The loaded AuNPs promoted electron transfer by immobilizing more capture probes introduced by Au–S bonds. AuNPs also acted as a carrier to load HRP as a signal probe to form a sandwich structure. The excellent properties of electrode-modified composite nanomaterials and AuNPs are fully utilized to realize the cooperative amplification of the signal. Compared with the low sensitivity of most traditional methods for the detection of miRNA, the application of metal nanomaterials not only improves the electrical conductivity but also improves the sensitivity and specificity of the electrochemical detection miRNA sensor system. However, due to the complexity of the preparation of nanocomposites, the superposition of multiple signal amplification strategies will also increase the complexity of the operation, coupled with the need for enzymes, and increase the cost.

Recently, nanomaterials with enzyme-like properties have attracted wide attention, and some nanomaterials have similar characteristics to enzymes. Au NPs are often combined with other nanomaterials as nanoscale enzymes. Kong and co-authors developed a sensor for electrochemical detection of miRNA based on the bifunctional nanomaterial Mn_3_O_4_@AuNPs^74^ (Fig. 6B). The nanomaterials not only have the characteristics of glucose oxidase-like activity and a self-supply of O_2_ but also reduce the dissolved O_2_ to low concentrations of H_2_O_2_. As a result, electrochemical signals are produced in the presence of other nanomaterials with peroxidase-like activity, and not only increase the concentration of H_2_O_2_, they also promote the oxidation of peroxidase-like enzymes, produce a cascade effect, improve catalytic efficiency, and significantly enhance the electrochemical signals. Compared with the shortcomings of natural enzymes, such as environmental instability, high cost and low recovery,^75–77^ the emergence of nano-enzymes can solve these problems, and also improve the catalytic efficiency. The sensitivity of the electrochemical detection of miRNA has, therefore, been further improved. Besides pure nanomaterials, gold nanomaterials are often combined with DNA nanowalkers or hybrid chain reactions linked with targets to achieve signal amplification. However, it is also more cumbersome to combine walkers with complex nanocomposites, use appropriate electrochemical active molecules as electrical signals, and modify hybrid chain reactions to avoid non-specific recognition, probe aggregation, as well as degradation of analytical performance. Meng *et al.* developed a general electrochemical biosensor driven by DNA enzymes for the sensitive detection of miRNA based on the multiple signal amplification of HCR induced by Mn^2+^ and the DNA-walker.^78^ The direct binding of Au NPs with the DNA-walker not only improved the electrical conductivity but also increased the chance of hybridization. The direct use of output molecules in the electrochemical system overcame the complex process of material preparation and amplification and provided the advantage of simple manufacturing. This work has also been verified by clinical samples, providing a new possibility for improving the electrochemical detection of miRNA.

Au NPs can significantly improve conductivity and sensitivity, are easily functionalized to fix the probe, and also promote the possibility of the application of other nanomaterials. The enzyme-like properties of Au NPs also make it better to amplify the signal and improve the sensing performance. However, it should be noted that Au NPs also have some potential disadvantages, the most obvious of which is the high cost, hindering the clinical application of metal nanoparticles. Moreover, due to the high conductivity and high specific surface area of metal nanomaterials, non-specific aggregation may occur with other substances, resulting in signal interference, which requires the continuous optimization of the sensing system and the introduction of specific recognition molecules to solve the problem.

Similarly, silver nanoparticles (Ag NPs) are widely used in the field of electrochemical biosensors because of the advantages of easy signal amplification, strong electrocatalytic activity and high extinction coefficient. Ag NPs are easily oxidized and a clear amplified signal can be obtained at low potential, so they are suitable for electrochemical redox labeling.^79^ Ag NPs can also be used as nano-signal probes to improve sensitivity. For example, a novel ratio electrochemical biosensor based on Ag NPs has recently been developed for the detection of miRNA^80^ (Fig. 6C). The basic principle is to adopt the dual-signal sensing mode, one of which is used as the detection signal and the other as the reference signal.^81^ Using the ratio of the two signals to detect the target, the signal interference is greatly reduced and the reproducibility of detection is improved. In this study, Ag NPs-labeled hairpin DNA was hybridized with a capture probe. Because the electrochemical signal of MB remains stable, it can be used as a reference source. The number of hairpins marked by Ag NPs as a detection signal is positively correlated with the concentration of the target miRNA. This detection mode can reduce the error, thus reducing the interference of the background signal, leading to high sensitivity and high accuracy. In addition, DNA template silver nanoclusters (DNA/Ag NCs) have attracted much attention due to their excellent electrochemical properties. Zhao *et al.*^82^ discussed the ability of specific base sequences to enhance or weaken the electrochemical performance of DNA/Ag NCs and constructed an ultra-sensitive miRNA biosensor platform. The sensor reported the enhancement of the activity of Ag NCs by T-rich sequences by combining with HCR amplification technology to detect miRNA. Due to the combination of two amplification techniques, the detection limit of this sensor is an order of magnitude lower than that of the traditional sensor based on HCR amplification technology.

Although Ag NPs have good electrical conductivity, good catalytic performance and optical properties, these advantages allow Ag NPs to be used in the electrochemical detection of miRNA sensors and also show good application potential in other fields. However, there are still some problems in the practical application of Ag NPs in electrochemical nano-biosensors. As precious metals, the use of Ag NPs is costly. This also hinders the possibility of mass production of Ag NPs-based electrochemical biosensors. In addition, in some cases, Ag NPs may cause immune reactions or toxic problems, limiting their application as biosensors that require long-term implantation or contact with biological tissue. In future research, more attention should be directed to the optimization of the stability and biocompatibility of silver nanoparticles, as well as the cost of production.

The application of platinum nanoparticles (PtNPs) in the electrochemical detection of miRNA mainly depends on the peroxidase-like characteristics of PtNPs with good catalytic effect on H_2_O_2_ reduction, so that the electrochemical signal can be measured by combining this characteristic with the target. Chen *et al.* successfully developed an electrochemical biosensor with high sensitivity and specificity for the detection of miRNA-21 by fully using the catalytic ability of Pt@CeO_2_ NSs to H_2_O_2_.^29^ Using the target to trigger the reaction, the capture probe with the hairpin structure was opened, and the biotin at the end of the probe was activated. Through the biotin–avidin affinity interaction, Pt@CeO_2_ NSs were successfully fixed on the electrode surface to catalyze H_2_O_2_ to amplify the DPV signal. In this work, the double catalytic ability of Pt and CeO_2_ to H_2_O_2_ was used to amplify the signal.

However, simple Pt NPs are prone to aggregation, which leads to poor stability. To solve this problem, Peng *et al.* developed a novel sensor system for triple signal amplification using Pt-supported covalent organic framed nanospheres (Pt@COFNSs) integrated with the electrochemical–chemical–chemical redox cycle and a target-induced magnetic DNAzyme walker as efficient catalysts^83^ (Fig. 6D). In this study, Pt nanoparticles were uniformly grown on the surface of COF to form stable nanocomposites, which improved the dispersion and stability of the material and displayed excellent catalytic activity. After that, the signal was significantly enhanced. Through these signal amplification methods, the detection limit was as low as 47.5 aM.

Although PtNPs have good electrical conductivity and catalytic activity and can enhance the signal well, they also suffer from high costs and the need to combine PtNPs with other nanomaterials due to the ease of aggregation of simple Pt nanoparticles, which increases the complexity of the operation. However, it seems that PtNP particles are very promising for electrochemical miRNA biosensing applications, greatly improving the performance of electrochemical biosensors.

### 5.2 Application of electrochemical miRNA biosensors based on two-dimensional nanomaterials

Compared with other types of nanomaterials, two-dimensional (2D) nanomaterials show superior electronic properties, higher specific surface area and surface energy, as well as good light transmittance and flexibility.^84^ Because the electrons of 2D nanomaterials are confined to the 2D plane, they show excellent electronic properties.^85,86^ With good light transmittance and flexibility and high specific surface area, they can provide more fixed sites, so they are ideal sensor application materials. Because the surface atoms of 2D nanomaterials are highly exposed, they are easily modified and regulated to show better performance and achieve effective signal amplification. There are a variety of synthesis methods, which provide greater possibility and controllability for the application of 2D nanomaterials. Compared with the difficulties of device integration and miniaturization faced by other dimensional nanomaterials,^87–89^ 2D nanomaterials show good compatibility with metal electrodes with larger transverse sizes. These unique advantages of 2D nanomaterials make them ideal materials for biosensors.

**Fig. 7:**
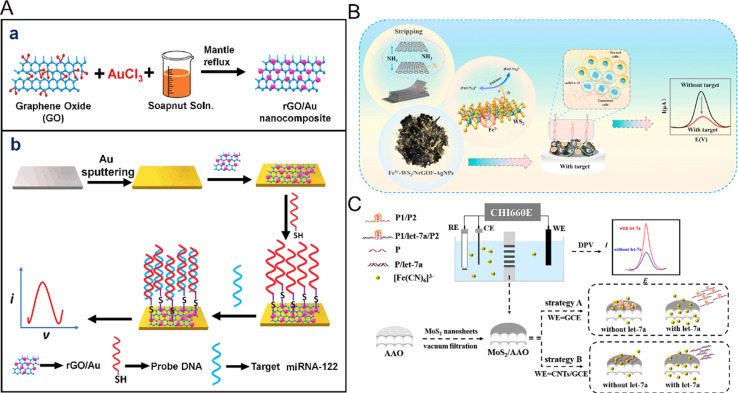
(A) Schematic representation of (a) the synthesis of the rGO/Au nanocomposite, and (b) the fabrication of the rGO/Au nanocomposite-based miRNA-122 electrochemical detection platform.^94^ Copyright 2020, Elsevier. (B) The electrochemical signal amplification strategy based on trace metal ion-modified WS_2_ for the ultra-sensitive detection of miRNA-21.^97^ Copyright 2023, Elsevier. (C) Illustration of the biosensing platform with MoS_2_/AAO as the recognition interface.^98^ Copyright 2023, Elsevier.

Single-element 2D nanomaterials mainly refer to 2D nanomaterials composed of single elements.^90,91^ Graphene, as the most representative single element 2D nanomaterial, is a new type of sp^2^ carbon atom sheet with single-atom thickness, which has attracted wide attention because of its inherent properties such as good electrical conductivity, high theoretical specific surface area, strong mechanical strength and good biocompatibility. Graphene is also conducive to the construction of miRNA electrochemical biosensors and can increase the electroactive surface area, and also enhance electron transfer and improve the adsorption rate of molecules on the surface. Asadi *et al.* developed a novel unlabeled electrochemical biosensor based on a graphene-modified glass carbon electrode using a molecular connector to fix the complementary probe DNA chain to the graphene-modified glass carbon electrode, which showed a high degree of stability and reactivity to the hybridization of the target miRNA-21.^92^ The electrochemical impedance method was used here to directly detect and quantify miRNA-21 with high detection sensitivity and a detection limit as low as 3 fM. Graphene oxide (GO) is not only a derivative of graphene, but also an important nano-material, which contains a large number of hydroxyl and carboxyl groups on its surface and has high dispersion in water, resulting in wide use in the detection of miRNA. The presence of surface hydroxyl and carboxyl groups makes GO sheets hydrophilic and also binds them to various types of inorganic nanoparticles, including metal oxides, semiconductor nanoparticles, precious metals and quantum dots (QDs), thus improving the performance of the sensor.^93^ For example, the composites of Au NPs and graphene are widely used as electrode materials for electrochemical biosensors because of their excellent electron transfer ability and large specific surface area. Kasturi *et al.* developed a novel high-resolution electrochemical biosensor^94^ (Fig. 7A) for the detection of reduced graphene oxide (rGO/Au) nanocomposites modified by miRNA-122 and gold nanoparticles. Nanocomposites were synthesized using natural soapnut solution as the reducing agent. The prepared reduced graphene oxide/gold nanocomposites were coated on the gold electrode, and the probe DNA was fixed on the binding site of the reduced graphene oxide/gold nanocomposites with a mercaptan joint, and the target miRNA-122 was identified with the electrochemical biosensor.

Graphene and its derivatives have a variety of excellent properties, good electrical conductivity, more active sites brought by high specific surface area, and so on. This makes the detection of miRNA more sensitive and rapid, and greatly promotes the development of electrochemical miRNA biosensors; however, there are still some shortcomings. The storage conditions and shelf life of graphene and its derivatives will affect the stability of the sensor. At the same time, the difference in different batches of materials is another problem that affects the stability of the sensor. Many conditions are needed to promote the development of electrochemical detection of miRNA. In addition to the excellent properties of the material itself, the cost and synthetic materials need to be considered. However, graphene and its derivatives still promote the development of electrochemical analysis of miRNA.

Besides graphene and its derivatives, transition metal disulfides, including WS_2_, MoS_2_, and WS_2_, have also received more attention due to their electronic tunability,^95^ low cost and good stability.^96^ For example, Fu *et al.* prepared floral nanocomposites of seven metal-doped modified WS_2_ by a simple one-pot hydrothermal method^97^ (Fig. 7B). 3D flower-like NrGOF was utilized as WS_2_ as the conductive interface, Fe^3+^ acted as a catalyst and electron transfer center for the secondary amplification of electrical signals, and silver was also mixed to increase the recognition sites. Based on this ultra-highly conductive nanoflower composite, an electrochemical biosensing platform for ultrasensitive detection of miRNA was constructed. Although this study proposed a high-performance nanocomposite electrochemical biosensor, there is a lack of in-depth study of the mechanism of action of metal nanomaterials, and the doping of multiple metals will also bring potential biotoxicity. MoS_2_ nanowires can not only quench fluorescence but also have a high specific surface area, which has broad prospects in the electrochemical detection of miRNA. Su *et al.* developed another novel electrochemical bioassay platform for MoS_2_ nanowires supported by anodic aluminum oxide film^98^ (Fig. 7C). MoS_2_ nanosheets showed unique adsorption capacity for single-stranded DNA and double-stranded DNA. The MoS_2_ nanosheets on the anodized aluminum film were used as an effective detection interface. Two strategies were proposed, namely, introducing the G-quadruplex and modifying the electrode, respectively. The purpose of detecting MoS_2_ was achieved by making use of the difference in physical adsorption between miRNA and single-stranded nucleic acid or pre-hybrid double strands. The joint action of the two strategies improved the detection range of miRNA, reduced the detection limit, and made the sensor system possess excellent selectivity. Compared with other nanomaterials, MoS_2_ has the characteristics of low cost and strong stability. By combining with other nanomaterials, it is possible to achieve mass production for the electrochemical detection of miRNA biosensors.

**Fig. 8:**
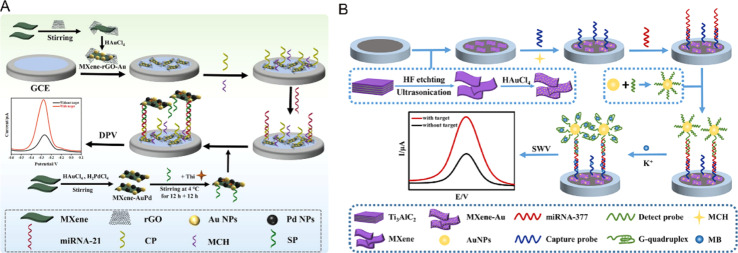
(A) Schematic diagrams of the preparation of MXene–rGO–Au nanocomposites and miRNA-21 electrochemical biosensors.^101^ Copyright 2023, Elsevier. (B) A schematic illustration of the design and fabrication of the electrochemical biosensor.^102^ Copyright 2022, Elsevier.

Recently, MXenes, which are new 2D nanomaterials and mainly include transition metal carbides, nitrides and carbonitrides, have been attracting increasing attention. They have been widely used in the field of biosensors because of their high conductivity, easy modification and functionalization, good hydrophilicity, easy preparation, and so on.^99,100^ As shown in Fig. 8, Guo *et al.* designed a sandwich biosensor based on MXene–rGO–Au nanocomposites, using MXene–AuPd as the signal amplification material.^101^ The MXene loaded on the electrode surface provides a large surface area for the fixed capture probe and promotes electron transfer. Coincidentally, Wu *et al.* reported an ultra-sensitive electrochemical biosensor based on MXene–Au nanocomposites and a G-quadruplex nano-amplification strategy.^102^ Through the *in situ* growth of Au NPs in MXene ultra-thin nanosheets, the prepared MXene–Au nanocomposites were used as substrate materials to fix the capture probe, and the signal was further amplified by the fact that the G-quadruplex could embed more MB. The integration of MXene materials and their nanocomposites with electrochemical sensors has great potential to improve the analytical performance of biosensors. Electrochemical biosensors based on MXene materials have been widely used in the field of miRNA detection.

As an emerging nano-material, these excellent properties provide MXenes with wider application prospects due to the excellent properties of MXenes, which are 2D nano-materials with large specific surface area and good electrical conductivity. However, the electrochemical miRNA sensors based on MXenes also face some problems such as complex material synthesis and low detection sensitivity. More convenient synthesis methods need to be developed and combined with other nanomaterials to improve their properties.

### 5.3 Applications of electrochemical miRNA biosensors based on nanotube materials

There are many types of nanotube materials, including carbon nanotubes (CNTs), boron–nitrogen nanotubes (BNNTs) and carbon–nitrogen nanotubes (CNNTs). Although BNNTs have good thermal stability and chemical properties, their electrical insulation limits their application in electrochemical biosensors.^103^ CNNTs exhibit semiconductor behavior and have few applications in electrochemical biosensors.^104^ Carbon nanotubes (CNTs) are the main materials for the electrochemical detection of miRNA. CNTs have been widely studied and applied in the construction of electrochemical biosensors because of their small size, hollowness, large specific surface area to volume ratio, high electrical conductivity and good biocompatibility. In electrochemical sensors, CNTs can be used as a fixed probe of a modified electrode and also promote electron transfer.

**Fig. 9:**
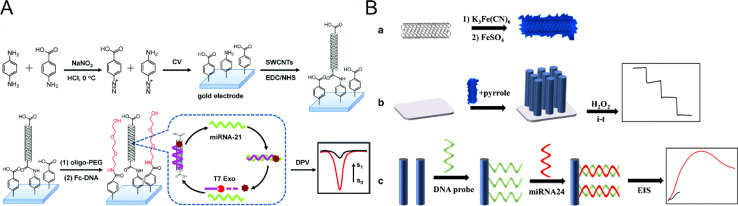
(A) Schematic illustration of the preparation process of the vertically aligned SWCNT-modified electrode.^105^ Copyright 2019, Royal Society of Chemistry. (B) Schematic preparation of MWCNTs/PB nanocomposites and illustration of the PPY/MWCNTs/PB nanowire array.^106^ Copyright 2021, Springer Nature.

Single-walled nanotubes (SWCNTs) and multi-walled carbon nanotubes (MWCNTs) are widely used in electrochemical sensors because of their excellent characteristics. For example, Chen *et al.* developed a new method for the determination of miRNA-21 by combining a one-step biometric reaction on a vertically aligned SWCNTs-based biosensor with T7 exonuclease (EXO)-assisted target recovery (Fig. 9A).^105^ The vertically arranged SWCNTs could achieve the non-covalent adsorption of ferrocene-labeled single-chain signal DNA; therefore, the construction of the sensor not only greatly improved the signal-to-target ratio of signal amplification, but also ensured the convenience of the method. The addition of vertically aligned SWCNTs caused the sensor to have efficient biometric ability and also promoted electron transfer. The lower limit of detection could reach 3.5 fM with a wide linear range. MWCNTs have the advantages of good thermal conductivity, small diameter and large specific surface area, which can promote the development of sensors. Yang *et al.* developed an electrochemical sensor based on a multi-walled carbon nanotube/Prussian blue-functionalized polypyrrole nanowire array (PPY/MWCNTs/PB),^106^ which could detect hydrogen peroxide and 33.4 fM miRNA-24 as low as 1.7 μM (Fig. 9B). Combined with the characteristics of PPY nanowires (good conductivity and large specific surface area) and MWCNTs/PB (excellent catalytic performance and redox activity), the materials showed excellent catalytic activity for the reduction of hydrogen peroxide, which was conducive to the construction of a high-performance miRNA biosensor platform. For application in electrochemical biosensors, nanotube materials have the advantages of a large specific surface area and good conductivity. However, there are also some urgent problems to be solved, including the complexity of nanotube synthesis and difficult size control, which will also lead to an increase in manufacturing costs and batch differences. The synthesis methods of nanotube materials need to be continuously improved to prepare nanotube materials with higher purity and reasonable size to ensure the stability and biocompatibility of the materials *via* combining with other nano-materials. The electrochemical miRNA biosensors based on the above types of nanomaterials are compared in Table 1.

### 5.4 The latest developments regarding miRNA sensors based on electrochemical detection by nanomaterials

Electrochemical miRNA sensors based on nanomaterials have become a new research field. However, for mass production and practical application, there is still the need to reduce the cost and constantly improve their stability and selectivity. The discovery of more and more new nanomaterials provides the possibility for mass production and practical application. Many nanomaterials-based electrochemical biosensors for multiple miRNA detection have greatly improved the detection efficiency.^116^ The traditional detection process is tedious and requires professional personnel for operation and, therefore, it is not only time-consuming but also costly. As a result, hand-held or intelligent nanomaterials-based electrochemical biosensors have been developed to obtain readings based on smartphones.^117^ This portable and simple sensor device brings great possibility for the application of clinical diagnosis.

## 6 Summary and outlook

Herein, we have reviewed the promoting effects of nanomaterials on the development of electrochemical miRNA biosensors. In recent years, compared with traditional detection methods, many new electrochemical miRNA sensors with nanomaterials have been reported, indicating the increasing interest in these electrochemical sensors. Nanomaterials play an important role in improving the performance of electrochemical miRNA biosensors because of their unique physical and chemical properties, large specific surface area and strong electron transmission capacity, which can act as ideal platforms for fixed recognition probes. Nanomaterials can also amplify the electrochemical signals produced by miRNA, thus improving the sensitivity and selectivity of biosensors. However, although nanomaterials have been well applied and developed in the field of electrochemical miRNA biosensors, there are still many challenges. These include how to efficiently prepare and explore new nanomaterials to improve the sensing performance; the high cost of nanomaterials, which hinders the possibility of practical application; and the potential biotoxicity, which requires the development of safer nanomaterials. The innovation of signal amplification strategies and the design of nucleic acid amplification strategies also require professional knowledge and personnel. Although interdisciplinary research can promote development, it also brings some complex problems. Moreover, for electrical signal molecules, the high-cost labeling of oligonucleotides or the difficulty synthesizing nanomaterials may limit their large-scale application in horizontal clinical practice. All these challenges have to be faced and therefore, it is quite necessary to explore new nanomaterials and their potential applications in electrochemical miRNA biosensors through skillfully combining nanomaterials with signal amplification strategy to construct more sensitive and stable electrochemical miRNA biosensors. The intersection of disciplines also requires researchers to gain more knowledge. With the continuous development of nanotechnology, electrochemical technology, molecular recognition technology, surface immobilization technology and other related technologies, these problems will be solved in the future and the electrochemical miRNA biosensors will continue to develop and be widely used in various scientific and technological fields.

## Conflicts of interest

There is no conflict to declare.

## Supplementary Material
